# Is clonal haematopoiesis the missing link between lupus and cardiovascular disease?

**DOI:** 10.1093/rheumatology/keag330

**Published:** 2026-06-25

**Authors:** Aamir Shamsi, Chris Wincup, Charis Pericleous, Fareeha Tariq, Matthew Sadler, Lynn Quek, Daniel Bromage

**Affiliations:** Department of Cardiovascular Sciences, British Heart Foundation Centre of Research Excellence, School of Cardiovascular Medicine, Faculty of Life Sciences and Medicine, King’s College London, London, UK; Department of Rheumatology, Kings College Hospital, London, UK; National Heart and Lung Institute, Hammersmith Hospital, London, UK; Department of Rheumatology, Kings College Hospital, London, UK; Department of Cardiovascular Sciences, British Heart Foundation Centre of Research Excellence, School of Cardiovascular Medicine, Faculty of Life Sciences and Medicine, King’s College London, London, UK; Department of Haematology, School of Cancer and Pharmaceutical Sciences, King’s College Hospital, London, UK; Department of Cardiovascular Sciences, British Heart Foundation Centre of Research Excellence, School of Cardiovascular Medicine, Faculty of Life Sciences and Medicine, King’s College London, London, UK

**Keywords:** lupus, cardiovascular, haematopoietic, immunogenetics, biomarker

## Abstract

SLE is an established, independent risk factor for cardiovascular disease (CVD), most notably premature atherosclerotic cardiovascular disease (ASCVD). This association is thought to relate to chronic immune-mediated inflammation. Clonal expansion of haematopoietic stem and progenitor cells (HSPCs) with acquired mutations, but no evidence of a blood disorder, is known as clonal haematopoiesis of indeterminate potential (CHIP). CHIP is associated with a pro-inflammatory immune phenotype, driven predominantly by mutant myeloid cells, and is similarly strongly associated with ASCVD. When SLE and CHIP co-occur, there may be synergy of their canonical cellular and molecular inflammatory pathways or even convergence of pathways through shared mechanisms of immune dysregulation, which accelerate CVD. Furthermore, enrichment of HSPC clones carrying CHIP driver mutations has been observed in chronic inflammatory conditions, including SLE. In this review, we explore the emerging role of CHIP in the relationship between SLE and ASCVD, proposing it as a pathogenic nexus in a triangular inflammatory network. Defining these relationships will help early identification of high-risk individuals and facilitate therapeutic targeting of CHIP to mitigate ASCVD complications in SLE patients.

Rheumatology key messages

- SLE and CHIP are independently associated with increased cardiovascular risk through dysfunctional immune responses and chronic inflammation.
- SLE and CHIP have distinct and overlapping cellular and molecular pathways that could synergize or converge to amplify ASCVD.
- CHIP could prove to be a mechanistic link between SLE and ASCVD, which would have significant clinical implications.

## Introduction

SLE is a chronic systemic disease characterized by abnormal immune activation resulting in systemic inflammation and tissue damage, notably in the joints, skin and kidneys [1,2]. Cardiovascular (CV) manifestations develop in >50% of SLE patients during their disease course [3], with a significant risk of accelerated atherosclerotic CV disease (ASCVD), including coronary, cerebrovascular, and peripheral vascular disease. This risk is independent of conventional CV risk factors such as diabetes and smoking [4].

Clonal haematopoiesis of indeterminate potential (CHIP) occurs when mutations in haematopoietic stem and progenitor cells (HSPCs) arise, without evidence of a blood disorder [5], leading to clonal expansion. CHIP is conventionally defined by a variant allele frequency (VAF) of ≥2% [6], and is associated with age, affecting ∼10% of those >65 years [5]. CHIP disrupts mature leucocyte function, promoting a pro-inflammatory environment [7]. CHIP is strongly associated with incident cardiovascular disease (CVD), especially ASCVD, and worse prognosis [8,9]. CHIP has been identified in >10% of SLE patients and occurred >20 years earlier than in controls without SLE [10]. Despite this, the relationship between CHIP, SLE and CVD is unknown.

While CHIP has been implicated in several systemic autoimmune rheumatic diseases (SARDs), we will focus on SLE as an exemplar of high premature CV risk and systemic inflammation. Here, we describe the pathophysiology of CVD, focusing on ASCVD, in both SLE and CHIP, and explore whether CHIP contributes to CVD in patients with SLE. We highlight which cellular and molecular pathways could be implicated, and what remains to be explored.

## SLE and cardiovascular disease

SLE has a highly variable clinical presentation and is estimated to affect 3.4 million people globally [11], with a higher prevalence in women [12]. The disease is characterized by abnormal innate and adaptive immune responses, including the generation of autoreactive B-cells that generate hallmark autoantibodies. Type I IFN (IFN-I) also plays a key pathogenic role, with higher levels associated with disease activity [13].

One of the most important causes of SLE-related morbidity and mortality are CV complications [2]. Individuals presenting with severe SLE are at increased risk of developing CVD [14]. However, even patients with well controlled disease have a 3–4-fold increased risk of CV events and death [15]. While SLE is associated with various CV complications (Table 1), ASCVD is the best described, with multiple pro-inflammatory pathways implicated in endothelial damage and subsequent atherosclerotic plaque formation. These pathways reflect a complex interplay between adaptive and innate immunity perpetuating a cycle of inflammation and mirror the broader immunopathology of SLE in other organ systems [29]. While we have a growing understanding of these processes, the exact mechanisms are not yet fully defined.

**Table 1:** Prevalence and pathophysiology of cardiovascular conditions in SLE.

CV condition	Prevalence in SLE	Pathophysiology
Pericarditis	♦ 82% prevalence (post-mortem evidence of pericardial inflammation) [2] ♦ Most common CV manifestation but mostly asymptomatic	♦ Infiltration of mononuclear immune cells, oedema and thickening from fibrous material [16], likely mediated by immune complexes [17] ♦ Haematoxylin bodies (degraded nuclear material and autoantibodies) are specific for lupus pericarditis and sometimes present [2] ♦ IL-17 pathway could also be involved in disease development [18]
Myocarditis	♦ 3% to 9% prevalence with mortality rate of 20% in small studies [19]	♦ Immune complexes made up of IgG and complement C1q may be present in the vessel wall and perivascular areas, but histology is generally non-specific [2, 16]
Atherosclerotic disease	♦ High prevalence with 50-fold higher incidence of MI and 17-fold increase in CAD-related death [2, 20]. This includes young females who are less likely to have traditional CV risk factors. ♦ Subclinical disease (atherosclerotic plaque before symptoms/events) is three times more prevalent than in controls [21].	♦ Pro-athoergenic antibody production ♦ Immune complexes bind to immune cells to release pro-inflammatory mediators ♦ IFN-I mediated plaque formation through endothelial damage and dysfunction ♦ IFN-γ promotes vascular remodelling, plaque formation, and endothelial damage ♦ Tregs are decreased, leading to atherosclerosis ♦ NETosis-mediated local tissue inflammation and damage ♦ Monocyte chemokine recruit inflammatory cells to the endothelium Increase in oxidative stress *Please see Section 4 for more details*
Valvular disease	♦ Up to 75% prevalence [2], with only 18% of those affected are likely symptomatic and 1% to 2% need surgery [22]. ♦ Libman–Sacks lesions can be found in chronic SLE with higher disease activity and APS but has become rarer with steroid treatment [23, 24].	♦ Deposition of complement and autoantibodies within the valvular structure can lead to valve thickening and regurgitation, with stenosis rarely seen, as well as appearance of Libman–Sacks vegetations (sterile growths) [25].
Heart failure/ cardiomyopathy	♦ 5× higher prevalence compared with controls [26], and 38% attributed to myocarditis [16].	♦ RIPK3, a component of the necroptosis pathway, is associated with cardiomyocyte hypertrophy, with RIPK3 inhibitor reducing diabetes-induced myocardial fibrosis [27] ♦ NLRP3 is upregulated in PBMCs in SLE and higher levels of NLRP3 inflammasome in DCM with NLRP3 inhibitor MCC950 reducing oxidative stress and cardiac dysfunction [27] ♦ Treatment with HCQ or chloroquine carries risk of cardiotoxicity
Arrhythmia	♦ Congenital heart block was found in neonates born to mothers with SLE and Ro positivity [2].	♦ Limited data available, but treatment with HCQ or chloroquine carries risk of fatal arrhythmias by prolongation of QT interval [28]

CAD: coronary artery disease; CV: cardiovascular; DCM: dilated cardiomyopathy; MI: myocardial infarction.

## Chip and inflammatory conditions

**Figure 1:**
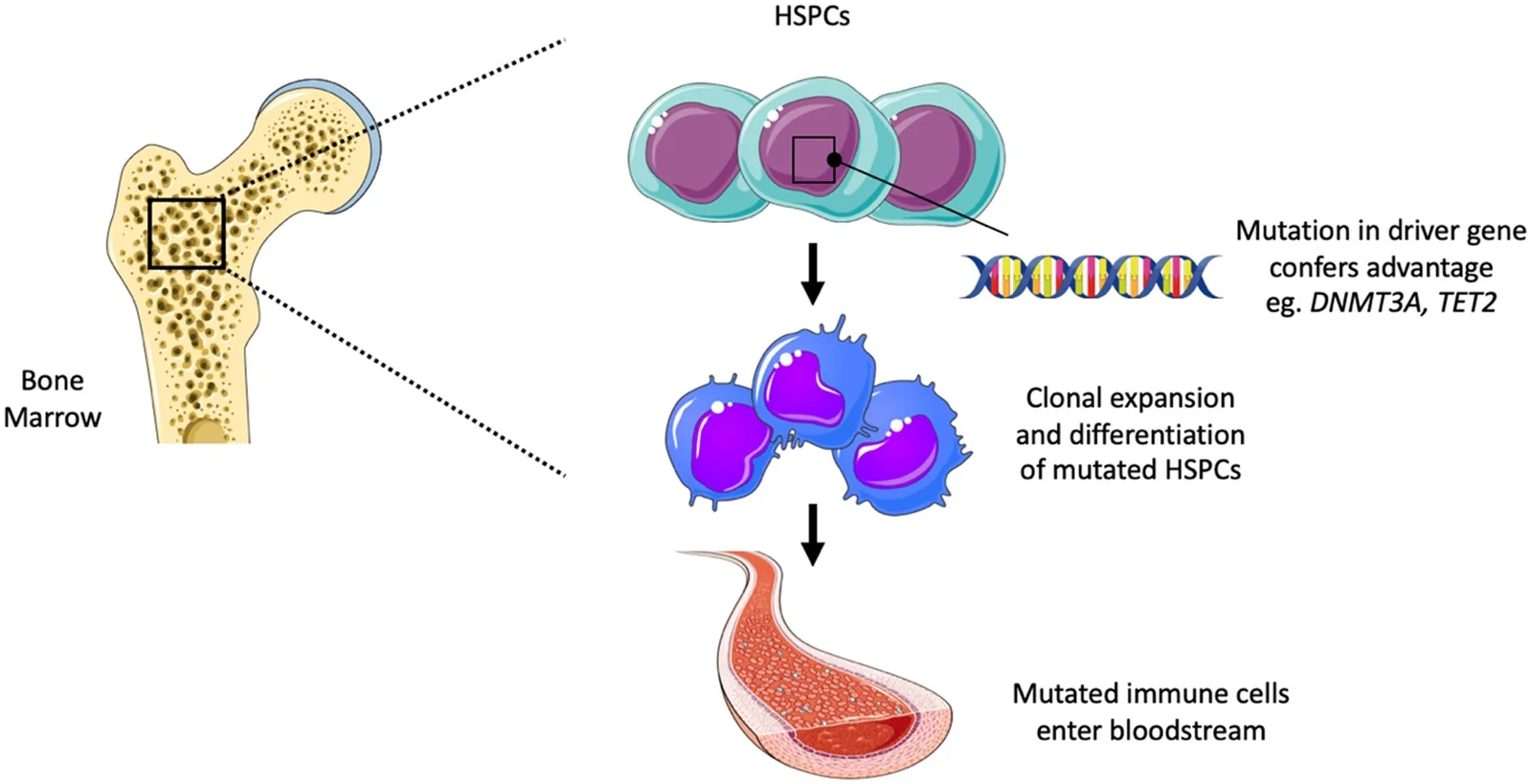
Clonal haematopoiesis at a glance. HSPCs: haematopoietic stem and progenitor cells. Image adapted from Servier Medical Art (https://smart.servier.com/), licensed under CC BY 4.0 (https://creativecommons.org/licenses/by/4.0/)

Clonal haematopoiesis (CH) occurs when somatic mutations in HSPCs result in a competitive growth advantage, leading to clonal expansion and reduced stem cell pool diversity (Fig. 1) [5]. When clones harbour driver mutations with a VAF of ≥2% and no evidence of myeloproliferative neoplasm (MPN), this is known as CHIP [6]. CHIP is an age-related condition with mutations detectable in 10% to 20% of those aged >70 compared with ∼1% of individuals <50 years [5]. CHIP is primarily detected from whole blood or peripheral blood mononuclear cell (PBMC) DNA via either Whole Exome/Genome Sequencing (WES/WGS), which provides a broad, lower-depth overview for population studies, or Next-Generation Sequencing (NGS) using high-depth targeted gene panels with error-correction allowing reliable detection of clones with a VAF of ≥2%. Most CHIP mutations are loss-of-function mutations of the epigenetic regulators *DNMT3A* (most commonly the R882 hotspot), a gene encoding a DNA methyltransferase, and *TET2,* encoding a methylcytosine dioxygenase [5]. CHIP mutations typically affect the myeloid lineage, and are associated with inflammation via increased expression of IL-1β, IL6, IL8 and NLRP3, among others [7]. Despite *DNMT3A* and *TET2* having opposing enzymatic activity on DNA methylation, the mutations have a similar hyperinflammatory phenotype, suggesting DNA methylation homeostasis protects the cell from a disease state, or other non-catalytic functions [30].

CVD is widely considered a chronic inflammatory condition. There is growing evidence of an association between CHIP and several CV conditions (Table 2), particularly ASCVD. Common mutations, such as *DNMT3A* and *TET2,* are associated with increased risk of incident coronary events [31]. Similarly, the less frequent *JAK2^V617F^* is associated with coronary artery disease (CAD) in case–control studies using WES [31]. This is supported by *TET2*-CHIP mouse models, which result in accelerated atherosclerosis [31, 54]. While ASCVD has been associated with low VAF (0.5% to 2%) [55], the greatest risk is seen with a VAF of ≥10% [31]. Mechanistically, both experimental and observational data in CHIP and ASCVD indicate a pro-inflammatory profile, supporting the notion that CHIP-related inflammation is underlying the disease pathogenesis.

**Table 2:** Prevalence and pathophysiology of cardiovascular conditions in CHIP.

CV condition	Prevalence in CHIP	Pathophysiology
Atherosclerotic disease	♦ 1.9-fold greater risk of developing CAD [31], and odds ratio of 4.0 for early-onset MI [31] ♦ 58% increased risk in PAD [32]	♦ IL cytokines promote necrotic atherosclerotic lesions and plaque instability ♦ Increased IFN-γ levels, which are known to contribute to atherosclerosis ♦ Tregs are inhibited, increasing ASCVD risk ♦ NETosis leads to endothelial inflammation, arterial thrombosis, non-calcified plaque burden and plaque instability ♦ Monocyte chemokines recruit inflammatory cells to the endothelium *Please see Section 4 for more details*
Valvular disease	♦ 1 in 3 patients with severe AS had CHIP in a small study [33]. ♦ CHIP in severe AS associated with an increase in mortality, despite successful intervention [34].	♦ Skew towards T-cells carrying *DNMT3A* mutation in CH-associated severe AS, whereas in *TET2*-CHIP–associated severe AS non-classical monocytes were majority mutant carriers [33].
Heart failure/ cardiomyopathy	♦ CHIP associated with 25% increase risk of incident HF [35], mostly related to those of <60 years. [36] ♦ CHIP has potential pathogenic role in HFpEF [37]. ♦ Higher CHIP detected in non-ischaemic DCM, specifically in those <30 years compared with the general population. [38] ♦ CH prevalence higher in non-ischaemic cardiomyopathy patients with cardiogenic shock compared with those with stable disease [39]. ♦ HF-related hospitalization and mortality associated with CH irrespective of aetiology [40], with a direct relationship between CH clone size and worse prognosis in ischaemic HF [41]. ♦ Immunosuppressive therapy has shown benefit in inflammatory cardiomyopathy trials [42].	♦ *TET2* associated with worse late-stage cardiac remodelling and reduced cardiac function after MI via NLRP3 inflammasome/IL-1β, possibly through migration of monocytes into the myocardium. NLRP3 inhibitor prevented heart failure [43]. ♦ *DNMT3A* deletion increased hypertrophy, fibrosis and reduced cardiac function via activation of cardiac fibroblasts [44]. ♦ *DNMT3A* mutation increased expression of T-cell–stimulating immunoglobulins via CD2 and altered skew to pro-inflammatory Th cells. [7] ♦ *ASXL1* mutation in germline DCM-mice prevented LVRR through infiltration of macrophages into myocardium to promote inflammation via IL-1 and IL-6 [45]. ♦ Increased mTORC1, known to stimulate hypertrophy, impair autophagy and worsen CVD. [46] Luspatercept, an mTORC1 modulator, reduced hypertrophy, LV end diastolic volume and serum BNP levels in *TET2*-CH [46].
Chemotherapy-related cardiac dysfunction	♦ Chemotherapy-related CH has been associated with the development of cardiac dysfunction [47]. ♦ Therapy-related CH (t-CH) differs from CH by enabling the survival of HSPCs under genotoxic stress rather than stimulating proliferation and self-renewal [47]. ♦ Increased CH, physiological ageing and CV mortality seen in childhood cancer survivors [47].	♦ Childhood cancer survivors showed a significant enrichment of DDRp mutant clones compared with *DNMT3A, TET2*, or *ASXL1* genes [47]. ♦ t-CH promotes non-ischaemic heart failure in mice through IL-1β inflammation [48]. ♦ Doxorubicin promoted greater Trp53-mutant neutrophil infiltration of the myocardium, leading to more ROS and pro-inflammatory cytokines. [49]
Inflammatory Myocardial and Pericardial Syndrome (IMPS)	♦ CHIP associated with 70% increased risk of IMPS with DNMT3A and TET2 conferring this risk [50] ♦ There is a VAF exposure–response gradient, ie. higher VAF levels associated with higher risk [50] ♦ Incidence remains unchanged when adjusted for any incident CV event, and association persisted if patients with known CVD were included, suggesting CHIP could be an independent risk factor [50]	♦ Mechanistic studies have implicated inflammasomes (eg. NLRP3) and pro-inflammatory cytokines (eg. IL1β and IL6) in development of CVD in CHIP, pathways which are also known to cause IMPS [50]. ♦ Inhibition of IL1β and IL6 can reduce severity of experimental myocarditis [50]. ♦ Randomization trials have shown benefit of IL-1–targeted therapies in recurrent pericarditis treatment [50].
Arrhythmia	♦ CHIP has also been associated with arrhythmias independent of CAD or HF [51]. ♦ AF patients with CHIP have more advanced disease, including development of HF [52].	♦ *TET2* mutations increased risk of incident AF regardless of CAD, through abnormal sarcoplasmic reticulum cardiac calcium release, and NLRP3 inflammasome activity and AF susceptibility was prevented by *NLRP3* inactivation [53].

AF: atrial fibrillation; AS: aortic stenosis; ASCVD: atherosclerotic cardiovascular disease; CAD: coronary artery disease; CH: clonal haematopoiesis; CHIP: clonal haematopoiesis of indeterminate potential; CV: cardiovascular; CVD: cardiovascular disease; DCM: dilated cardiomyopathy; HF: heart failure; HFpEF: heart failure with preserved ejection fraction; HSPC: haematopoietic stem and progenitor cell; LVRR: left ventricular reverse remodelling; MI: myocardial infarction; mTORC1: Mechanistic Target of Rapamycin complex 1 activity; PAD: peripheral artery disease; ROS: reactive oxygen species; VAF: variant allele frequency.

However, the directionality of this association in humans remains debated. While a recent observational study suggests CHIP drives ASCVD [56], other work indicates ASCVD, as well as concomitant cardiovascular risk factors that precipitate chronic low-grade inflammation (such as obesity, diabetes and smoking [30, 57, 58], promotes HSPC mutant clone expansion [59]. Murine studies [60, 61], corroborated by human genome studies [62], have shown that increased circulating pro-inflammatory cytokine levels are associated with higher VAF and increased clonal fitness, resulting in a harmful cycle sustaining and exacerbating inflammation [59]. This suggests the relationship between CHIP and ASCVD is complex, involving bidirectionality and positive feedback loops, and may explain why even small clones perpetuate inflammation leading to ASCVD.

Notably, chronic systemic inflammation is a hallmark of SLE and, as with CHIP, underlies its association with CVD. However, the role of CHIP in SLE and other SARDs remains under-investigated [63]. In a SSc cohort aged <50 years, CHIP is higher in cases compared with controls (25% *vs* 4%), but is not associated with adverse outcomes [64]. CHIP was also higher in ANCA-associated vasculitis (AAV) patients *vs* age-matched controls, with nearly a fifth of cases in those aged ≤55 years [5, 65]. Another study showed CH was detected in 34% of a patient cohort of AAV, giant cell arteritis (GCA) and Takayasu’s Arteritis (TAK) across the age spectrum compared with 18% of age-matched controls, with clinical relapse in GCA correlated to CH in a dose-dependent manner [66]. A further study shows that CHIP prevalence was 17% in RA, increasing to 25% if aged 70–79 years [67].

Only one study has investigated CHIP in SLE specifically. In the HEMATOPLUS study of 438 SLE patients with a median age of 38 years, 10.7% have CH, increasing to 26.3% among those aged 50–59 years [10]. Interestingly, CHIP frequently occurs before the age of 60 and >20 years earlier than in controls [10], suggesting SLE promotes early-onset CHIP. *DNMT3A* was the most commonly mutated gene, consistent with other CHIP studies. Interestingly, CHIP prevalence is not associated with the use of immunosuppressive therapy [10], but is associated with a less severe SLE phenotype, based on less renal involvement. There is also no increase in incident CVD in the CHIP group. This may be explained by the low baseline CV risk, relatively low mean VAF and incomplete reporting of CHIP details and incident CV events [10]. The HEMATOPLUS findings contrast with the WES study that identifies an association between *DNMT3A* mutations and a severe SLE disease phenotype with nephritis [68]. This discrepancy is potentially explained by patients in HEMATOPLUS being older and new-onset LN being less common than in older SLE patients.

Therefore, the association between CHIP and SLE requires further study. Consistent with other chronic inflammatory conditions, SLE has the potential to promote mutant clonal expansion, particularly given that SLE creates an inflammaging phenotype and CHIP is an age-associated phenomenon. There is also evidence to suggest that CHIP facilitates the inflammatory environment characteristic of SLE, potentially contributing to its development or progression. Any such association would also amplify CV risk and could explain why, beyond solely disease activity, young female SLE patients, who are less likely to have traditional CV risk factors, have a significant risk of myocardial infarction (MI) [20] Chronic inflammatory diseases, particularly SARDs, offer important mechanistic insights into the interplay between CHIP, chronic inflammation, and ASCVD. Elucidating this link could help risk-stratify patients, tailor anti-inflammatory regimens, and identify novel therapeutic targets.

## Mechanistic insights linking chip, SLE and CVD

Here, we explore mechanisms that may underlie a causal link between SLE, CHIP and CVD, and shift the perspective from viewing SLE and CHIP as independent bystanders towards a biological relationship.

### Synergy of distinct inflammatory axes

**Figure 2:**
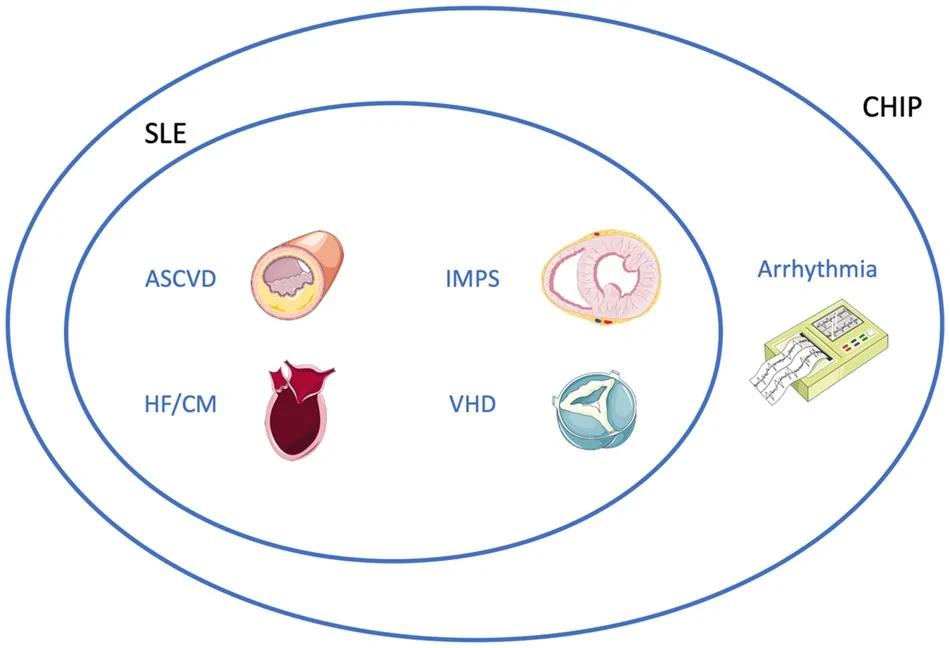
Cardiovascular conditions associated with SLE and CHIP. ASCVD: atherosclerotic cardiovascular disease; CHIP: clonal haematopoiesis; CM: cardiomyopathy; HF: heart failure; IMPS: inflammatory myocardial and pericardial syndrome; VHD: valvular heart disease. Image adapted from Servier Medical Art (https://smart.servier.com/), licensed under CC BY 4.0 (https://creativecommons.org/licenses/by/4.0/)

SLE and CHIP are independent CVD risk factors (Fig. 2), which confer risk through dysregulation of largely distinct aspects of the immune system, resulting in chronic inflammation that promotes atherosclerosis. For SLE, this is via both altered B cell biology and downstream crosstalk with innate immune cells, whereas CHIP is typically associated with aberrant myeloid cell biology. However, when they co-exist, there could be synergy of these axes towards accelerated ASCVD.

#### The lupus–cardiovascular axis

The two pathognomonic hallmarks of SLE are loss of B cell tolerance and Type I IFN (IFN-I) cytokines. Aberrant B-cells produce autoantibodies directed against nuclear components, including ANA, anti-dsDNA antibodies and anti-Sm antibodies. These autoantibodies form immune complexes with self-antigens, for example oxidized low-density lipoproteins (oxLDLs) [69], which bind to receptors on immune cells and activate NF-κB, leading to the release of downstream pro-inflammatory and vascular cell adhesion molecules. This results in the further recruitment of pro-inflammatory immune cells, tissue damage, and, ultimately, clinical manifestations of the disease [23, 70]. Immune complexes in SLE exhibit a pro-atherogenic role [23, 70]. Furthermore, athero-protective antibodies, such as anti-apolipoprotein-B, are reduced in SLE [71], and dysfunctional regulatory B-cells are unable to promote tolerance or suppress excessive inflammation [72]. aPLs in particular have been noted to trigger thrombosis and MI, through platelet and endothelial activation, even in the absence of plaque [16].

Immune complex binding to immune cells also activates IFN-I production [73], most commonly by plasmacytoid dendritic cells and other innate immune cells. IFN-I has broad immunomodulatory functions, including priming innate immunity, dysregulating T cell function, and promoting activation and expansion of autoreactive B-cells, creating a positive feedback loop [74–76]. IFN-I levels are not only associated with SLE disease activity [13], but also higher coronary calcification and endothelial damage, which increases plaque formation [70]. IFN-α, an IFN-I, disrupts endothelial nitric oxide (NO) synthase expression and impairs NO signalling, which is needed for inhibiting platelet aggregation and endothelial leucocyte adhesion [77]. IFN-I also suppresses production and maturation of endothelial-like progenitor cells, which represent a myeloid angiogenic population capable of paracrine-mediated endovascular repair after injury [78,79]. IFN-I also increases expression of macrophage scavenger receptor class A that recruits macrophages to uptake oxLDLs and form foam cells [80], further driving plaque formation. Consistent with this, anti-IFN-I receptor antibodies improved cardiac function and survival post-MI in a mouse model [81].

SLE therapy also increases ASCVD risk. For example, glucocorticoids are associated with hyperlipidaemia, hyperglycaemia and hypertension, with prolonged use in SLE significantly linked to CVD [82]. Likewise, AZA and ciclosporin have been implicated in atherosclerosis [83] In summary, chronic inflammation driven by immune crosstalk in SLE leads to endothelial dysfunction and injury, resulting in ASCVD.

#### The bone marrow–cardiovascular axis

Studies of CH-associated ASCVD demonstrate upregulated pro-inflammatory pathways. Here we review the best understood pathways, including inflammasome activation, and IL-1 and IL-6 cytokine cascades, referring to both human and animal models, although the latter do not closely mimic human CHIP driver mutations or diversity.

First, increased IL-1β driven by inflammasome activation is seen in CHIP models. A large study of mixed cohorts found increased IL-1β in *TET2*-CHIP [62]. Similarly, *TET2*-deficient mice show upregulation of macrophage-derived pro-inflammatory cytokines, including IL-1β, with levels exceeding transcriptional activation, suggesting additional post-transcriptional inflammasome enhancement [43]. Furthermore, IL-1β promotes recruitment of wildtype leucocytes into atherosclerotic plaques [54]. In *TET2*-CHIP mice, the NLRP3 inhibitor MCC950, that disinhibits NLRP3 inflammasome, reversed atherosclerosis [54]. Similarly, blocking BRCC3, a protein involved in DNA repair and activation of NLRP3, improved plaque stabilization [84]. The NLRP3-IL-1β pathway is thus an important modulator of *TET2*-CHIP–associated ASCVD.

IL-1 signalling comprises several cytokines, some independent of inflammasome activation. IL-1 mRNA expression is increased in low-VAF mutant *JAK2^V617F^* macrophages compared with control mice, promoting necrotic atherosclerotic lesions and plaque destabilization [85]. Mechanistically, increased IL-1 cleaves macrophage receptors MerTK, which reduces the necrotic core of lesions, and TREM2, which promotes efferocytosis and cholesterol efflux [85]. Notably, IL-1 receptor knockout in *JAK2*-CHIP mice stabilize these receptors and improve plaque stability [85], suggesting a role for IL-1 in both disease progression and immune crosstalk. Given the prominent role of IL-1 in *JAK2*-CH, inflammasome activation is expected. As predicted, *JAK2*-CHIP mice demonstrate increased AIM2, of the AIM2 inflammasome, while its deficiency or deletion of essential inflammasome components reduced atherosclerosis [86].

Other inflammatory cytokines are also implicated in CHIP-mediated ASCVD*. TET2*-CHIP macrophages in a UK Biobank study showed IL-6, downstream of IL-1β, modulates disease development, as *TET2*-CHIP individuals carrying dysfunctional IL-6 receptors had a pronounced reduction in ASCVD risk [87]. *DNMT3A-*CHIP models also augment monocyte pro-inflammatory cytokine expression, such as IL-1β and IL-6 [7, 88].

In summary, the bone marrow– and lupus–cardiovascular disease axes comprise distinct pathways leading to ASCVD that, when co-activated, potentially synergize to exacerbate disease.

### Convergence of shared inflammatory pathways

**Figure 3:**
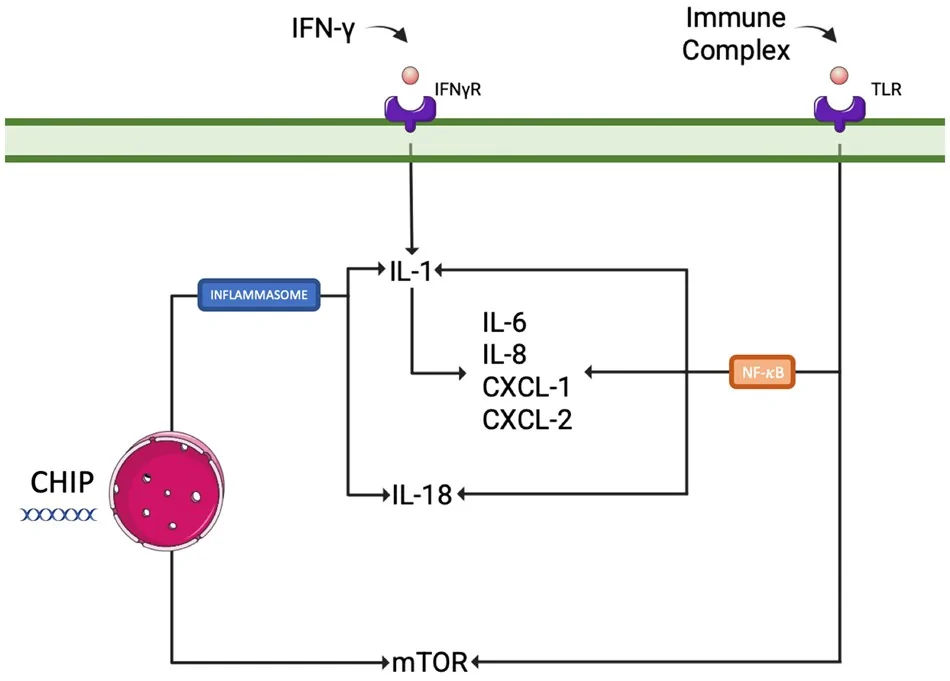
Shared cytokine, chemokine and dysregulated metabolic pathways between SLE and CHIP that lead to ASCVD. ASCVD: atherosclerotic cardiovascular disease; CHIP: clonal haematopoiesis; IFNγR: IFNγ Receptor; mTOR: Mechanistic Target of Rapamycin; TLR: Toll-like Receptor Image adapted from Servier Medical Art (https://smart.servier.com/), licensed under CC BY 4.0 (https://creativecommons.org/licenses/by/4.0/)

Immune crosstalk is an important characteristic of both SLE and CHIP. In SLE this manifests as interaction between autoreactive and self-tolerant immune cells, while for CHIP there is communication between wildtype and mutant clones. This may explain why, despite SLE and CHIP having distinct cellular and molecular pathways that drive ASCVD, they converge on several shared pathways (Fig. 3). Therefore, when both co-exist, predominantly myeloid CHIP could converge with B cell–mediated SLE, and vice versa, creating further positive feedback loops and a vicious cycle of systemic chronic inflammation and compounded ASCVD risk.

#### Type I and II IFN cytokines

The NLRP3 inflammasome, central to *TET2*-CHIP–mediated ASCVD, can be activated by IFN-I [89], which, as described above, is a key pathogenic cytokine in SLE driving tissue damage and atherosclerosis. Inflammasome activation by parallel upstream pathways likely serve to augment its effects.

IFN-γ is a Type II interferon increased in both CHIP and SLE, largely from dysregulated CD4^+^ T cell signalling [1, 90]. In *DNMT3A* deficiency, there is increased productionof IFN-γ [91], promoting the activation of macrophages, generation of matrix metalloproteinases and increased expression of vascular cell adhesion molecule [2]. This leads to vascular remodelling, plaque formation, and endothelial damage [2]. In SLE, IFN-γ additionally drives aberrant B cell differentiation and autoantibody production. The atherogenic function of IFN-γ is modulated through the production of IL-1 in CHIP; in SLE, TNFα is also involved [92].

#### IL cytokines

IL-1, specifically IL-1β, is central in *TET2*-CHIP– and *JAK2*-CHIP–associated ASCVD, as described in the previous sections, but is notably also increased in SLE-associated ASCVD via NF-κB, where it activates platelets [93]. IL-6 and IL-8, which are activated downstream of NLRP3 inflammasome-IL-1β in CHIP models, and upregulated in SLE via NF-κB, contribute to ASCVD by promoting leucocyte recruitment to the endothelium and disrupting the vascular barrier [7, 87, 88, 94]. IL-18, another inflammasome and NF-κB product, which are upregulated in both *JAK2*-CH and SLE, can modulate atherosclerosis by promoting NETosis and plaque instability [95, 96]

#### Loss of protective T cell function

Tregs, which normally maintain self-tolerance and suppress excessive inflammation, are inhibited in *DNMT3A*-CH [97], and decreased in SLE [98]. This contributes to atherosclerotic lesions in an SLE mouse model [99]. Invariant natural killer T cells (iNKTs), which help differentiate macrophages to an atheroprotective phenotype, are unresponsive in SLE-associated ASCVD, further contributing to disease progression [100]. Although cytokines in CHIP-associated ASCVD pathways may influence iNKT activity [101], no studies directly implicate these cells.

#### NETosis

Low-density granulocytes (LDGs) release web-like products called neutrophil extracellular traps (NETs) through NETosis, a type of programmed cell death (PCD). In JAK2-CH, NETosis occurs downstream of IL-1 and cleaved MerTK [85, 102]. NETs perpetuate inflammation and are linked to endothelial damage, arterial thrombosis, non-calcified plaque burden and plaque instability [71, 102, 103]. NET formation is also increased in SLE, mediating local tissue damage and serving as a source of autoantigens [104].

NETs also activate adjacent macrophages leading to NLRP3 inflammasome activation and cytokine release in both murine CHIP and SLE models, with impaired NET function being associated with reduced atherosclerosis [95, 103]. By activating inflammasome function, a potential positive feedback loop develops, with IL-1 promoting further NETosis. This highlights how both the CHIP– and lupus–cardiovascular axes involve immune crosstalk and suggests convergence to amplify ASCVD risk.

While other types of PCDs, such as apoptosis, ferroptosis and necroptosis, occur in SLE-associated atherosclerosis, MI and ischaemia-reperfusion injury [105, 106], there is no direct evidence of their involvement in CHIP-associated ASCVD, though some pathway components overlap.

#### Monocyte chemokine expression

Myeloid lineage cells predominate in driving inflammation observed in CHIP-associated ASCVD. *TET2*-CHIP macrophages show increased CXCL1/2/3 expression mostly through IL-1β, [32, 43] and *DNMT3A-*CHIP models augment CCL3/4 [7, 88]. These chemokines recruit inflammatory cells to the endothelium, promoting damage and foam cell formation that drive ASCVD progression.

Similarly, pro-inflammatory monocytes increase and overactivate in SLE with clinical and subclinical CVD, but not in those without, highlighting overlapping inflammatory pathways in CHIP-associated ASCVD [70]. Macrophages produce pro-atherosclerotic mediators in SLE, through NF-κB, such as IL-1β and CXCL1/2, mirroring CHIP, as well as CCL2 [107]. Additionally, both M-CSF and neopterin, a monocyte/macrophage activation marker, are elevated in SLE and strongly associated with increased ASCVD risk [108, 109].

#### Metabolic dysregulation

CH-associated cardiac stress has been linked to Mechanistic Target of Rapamycin (mTOR) complex 1 activity [46] Cellular bioenergetics are disrupted through increased mTOR activity impairing mitochondrial function and overproducing reactive oxygen species (ROS), driving oxidative stress, cell death and increased CV morbidity [110]. Furthermore, CHIP directly increases oxidative stress, albeit in myeloproliferative disease. In SLE, impaired mitophagy is similarly described through increased mTOR activity [111], leading to the release of highly immunogenic material that perpetuate the immune response [112, 113].

Further metabolic dysregulation includes dyslipidaemic profiles. NETs, observed in CHIP and SLE, destabilize high-density lipoprotein (HDL), creating a pro-inflammatory and pro-oxidant state [114, 115], with levels correlating to CAD severity [71]. In SLE, very-low-density lipoprotein, a pro-thrombotic lipid, rises while typically atheroprotective HDL, falls [116].

Notably, *DNMT3A* is significantly raised in adipose tissue–derived macrophages that mediate insulin resistance [117], and *TET2-*CHIP mice showed increased insulin resistance and obesity [118]. SLE is similarly associated with insulin resistance, as well as increased serum leptin levels and metabolic syndrome, all increasing CV risk [119–121].

#### Immunosenescence

CHIP may create an inflammaging profile via chronic inflammation [122], exhausting HSPCs and promoting immune dysregulation [123]. This is further supported by CHIP linked to age-related conditions [57], exhibiting high circulating levels of inflammaging-associated IL-6 and TNFα [122], and a myeloid bias typical of senescence [124]. However, longer telomere length, which is associated with slower ageing, was shown to be a causal risk factor for CHIP in a Mendelian randomization analysis [125]. This was corroborated by bidirectional Mendelian randomization studies, which, interestingly, also show CHIP-accelerated telomere shortening [126]. This difference can be explained by longer telomeres conferring survival bias, and selecting for CHIP clones, which subsequently drive telomere attrition and senescence in other cells and tissues.

Metabolic dysregulation in SLE boosts immunosenescent (age-related dysfunction) CD4^+^ cells that are not only pro-inflammatory and resistant to apoptosis, but also associated with atherosclerosis [70, 110]. Other markers for immunosenescence seen in SLE include decreased phagocytic capacity and premature telomere erosion [110]. This inflammaging signature perpetuates chronic inflammation that results in clinical manifestations of SLE, including ASCVD. The relationship between CHIP and inflammaging remains complex and ill-defined but, if present, potentially overlaps with the immunosenescent profile of SLE to perpetuate chronic inflammation.

The shared pathways of CHIP and SLE discussed in this section drive inflammation and convergence would accelerate ASCVD.

### Triangular inflammation: is CHIP the pathogenic nexus?

We have highlighted how the co-existence of SLE and CHIP likely synergizes to amplify ASCVD via the complementarity of their inflammatory pathways, including dysregulated lymphoid cells in SLE and dysregulated myeloid cells in CHIP, as well as convergence and crosstalk of their respective pathways. However, this alone may not explain the heightened ASCVD risk. As described previously, despite the association of CHIP with various inflammatory conditions, there is currently limited evidence of its association with SARDs, including SLE. Nevertheless, there are some compelling observations that shed light on a potential bi-directional relationship, suggesting CHIP contributes to the pathogenesis or exacerbation of SLE, and SLE promotes CHIP. If there is causal relationship between SLE and CHIP, then the pathways we have described would not just act in parallel but, instead, form a connected triangular inflammatory network, substantially augmenting ASCVD risk.

The epigenetic dysregulation that underlies SLE primarily involves abnormal DNA methylation [127]. *DNMT3A* and *TET2* play a significant role in this process while also being key CHIP driver genes. Such CHIP mutations may create an environment wherein SLE develops. This is supported by *TET2* mutations being associated with autoimmunity, including SLE development, in patients with myelodysplastic syndrome [128]. Furthermore, a study of PBMCs from 101 SLE patients suggests epigenetic modification of *TET2* promoters could contribute to SLE development or disease progression [129]. *TET2* mutations are also known to increase IL-6 [87], and regulate IFN-I [30], both involved in SLE pathogenesis. Interestingly, NLRP3 inflammasome, which is prominent in *TET2*-CHIP, has been associated with NETosis [84], as well as IFN-I and autoantibody production through its component Caspase-1 [89], all core elements of SLE pathogenesis. Dysfunction of MerTK, observed in *JAK2*-CHIP, has also been associated with SLE risk [130], especially LN.

There is also some evidence of CHIP leading to SLE from mechanistic studies. A mouse model shows that dysregulated IL-21–TET2–AIM2–c-MAF signalling pathway drives CD4^+^ T cell response and autoantibody production in SLE pathogenesis [131]. Furthermore, wildtype AHR/TET2/NT5E axis downregulation in human Tregs is associated with the development of SLE and disease progression [132], suggesting loss-of-function mutations also contribute to SLE pathogenesis. Additionally, a murine model shows decreased *DNMT3A* mRNA expression induced anti-dsDNA antibodies [133], one of the hallmarks of SLE, and the JAK2/STAT3 pathway has been implicated in T cell impairment in lupus [134].

Conversely, there is an increased risk of myeloid neoplasms, which are associated with CHIP, among SLE patients [135]. This suggests that dysregulated inflammation in SLE, which is known to contribute to clonal expansion [136], primes the bone marrow for CHIP emergence. Furthermore, IFN-I can regulate HSPCs, with chronic signalling leading to activation and exhaustion, further promoting CH [137, 138]. Molecular products of SLE pathways, such as CCL2 and TNFα, also play a role in HSPC proliferation and conceivably provide a fitness advantage to mutant cells [139,140].

Additionally, SLE and CHIP share risk factors, implying common pathological mechanisms leading to immune dysregulation. The use of immune checkpoint inhibitors can induce both drug-induced lupus and CH [141,142]. Infection can act as a trigger to drive clinical manifestations of SLE [1] and promote CHIP through inflammatory stress [58] Smoking and consequent oxidative stress and DNA damage is also thought to be an environmental trigger for both SLE and increased clonal expansion in CH [1, 58].

**Figure 4:**
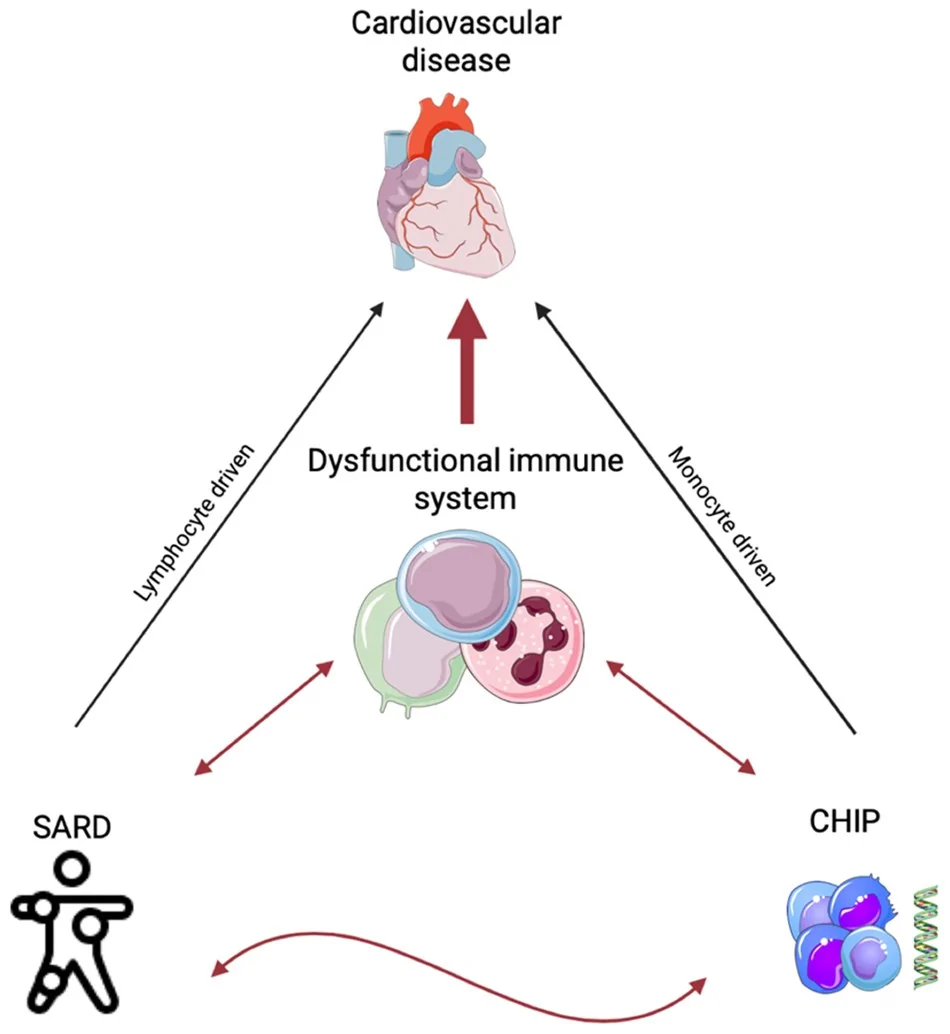
Suggested triangular inflammatory network between SLE, CHIP and CVD. ASCVD: atherosclerotic cardiovascular disease; CHIP: clonal haematopoiesis; CVD: cardiovascular disease; SARD: systemic autoimmune rheumatic disease. Image adapted from Servier Medical Art (https://smart.servier.com/), licensed under CC BY 4.0 (https://creativecommons.org/licenses/by/4.0/)

Uncertainty remains over whether the association between CHIP and SLE is the result of shared risk factors, incidental co-occurrence, or a bidirectional, causal relationship. If the latter is true, it suggests that, beyond synergy of SLE and CHIP-driven inflammation, CHIP could be a mechanistic bridge through which SLE patients go on to develop ASCVD and/or a mechanistic precursor that predisposes to both SLE and ASCVD. This would create a multidirectional, interconnected and triangular cascade that would intensify the inflammatory process to propel tissue damage and thus ASCVD, making CHIP a pathogenic nexus in the SLE–ASCVD continuum (Fig. 4). In line with this hypothesis, CHIP develops prematurely in SLE [10], and CV events occur on average 10 years earlier in this cohort than in the general population [143]. In addition, mutant *DNMT3A* has been associated with a severe SLE phenotype [68], while initial SLE severity is associated with higher ASCVD risk [14]. More work is needed to elucidate the role of CHIP in CVD pathogenesis for SLE patients, but here we propose CHIP acting as a driver for early CVD in SARD and SLE populations.

## Clinical implications

CHIP- and SLE-associated CVD are associated with worse prognosis. If a mechanistic link between SLE, CHIP and CVD can be proven, then identifying this cohort would be of significant clinical importance in re-classifying and managing patients, given the additive impact of both SLE and CHIP on premature CVD risk.

Combining primary genetic biomarkers, such as somatic mutations with a VAF of ≥2% in common/high-risk genes, with more accessible surrogate biomarkers, such as IL1β and IL6, which reflect the pro-inflammatory state found in CHIP [144], could help stratify those at higher CV risk. High-sensitivity CRP (hsCRP), a non-specific marker of inflammation, has been shown to be a strong predictor of CV events [145, 146], and could represent another surrogate biomarker for CHIP-driven systemic inflammation [144]. Utilizing these biomarkers may guide a more patient-centred and intense primary prevention strategy [144]. This includes closer follow-up and stricter control of CV risk factors, which has been shown to significantly reduce atherosclerotic progression in SLE [147].

Next, specific targeting of inflammatory pathways in CHIP carriers is already of growing interest and may offer therapeutic benefit in both primary and secondary prevention to interrupt the bone marrow–CVD axis, particularly in the context of SARD, especially given that there is limited evidence for disease-specific CV risk management and treatment in this cohort [148]. The CANTOS trial showed that canakinumab, an IL-1β monoclonal antibody, reduced IL-6 levels and CV events independent of lipid-lowering therapy in patients with stable CAD [149]. Notably, a sub-study showed these benefits persisted among *TET2*-CHIP patients, but not among those with *DNMT3A*-CHIP, suggesting a mutation-specific therapeutic response [150]. Similarly, colchicine, an anti-inflammatory agent that targets multiple cytokine pathways, including IL-1β, IL-6, IFN-γ and NLRP3 inflammasome, reduced IL-1β and atherosclerosis in a *TET2*-CHIP mouse model [151]. Real-world data has supported the beneficial role of colchicine in ASCVD, showing an association with lowered MI risk in *TET2*-CHIP patients [151]. Additionally, ruxolitinib, a JAK/STAT signalling inhibitor, has been shown to reduce IL-18 [152] and attenuate atherosclerotic plaque in animal models [153], offering another potential inflammation-modulating therapeutic target. Although the use of IL1 and JAK inhibition is not established in SLE therapy, they may still play a role in preventing or treating CHIP-associated CVD in SLE patients.

## Conclusions and future directions

SLE, a B cell–mediated SARD, and CHIP, which is mostly myeloid driven, create a chronic inflammatory environment and drive the development of ASCVD through their respective dysfunctional immune responses. Although SLE and CHIP are independent risk factors for ASCVD, they have overlapping cellular and molecular pathways that could synergize and converge to amplify risk when they co-exist.

We propose research to ascertain the link between SLE, CHIP and CVD to provide in-depth insights into the pathophysiology and intersection between autoimmunity, inflammation and CVD biology. A potential area of interest would be the role, if any, of B cell CHIP, which has been underexplored, particularly in the context of SLE and CVD. We also suggest robust longitudinal assessment of CV events in CHIP and non-CHIP SLE patients, and investigating subclinical CVD in cross-sectional studies. Of course, as CHIP has also been identified in other SARDs that also have a strong association with CVD, further work should not be limited to SLE.

If CHIP is proven to be an important risk factor for CVD in SLE patients, this would result in clinically meaningful improvements via intensification of CV risk factor control for those who carry this genomic mosaic. It may also open the door to targeted and personalized immunomodulatory therapy for this population. With advancements in genetic testing and integrated bioinformatics, identifying a biomarker for CHIP would help to significantly reduce CV complications for the SLE population.

## Data Availability

No new data were generated or analysed in support of this research.
